# Development and validation of artificial intelligence models for automated periodontitis staging and grading using panoramic radiographs

**DOI:** 10.1186/s12903-025-07025-8

**Published:** 2025-10-14

**Authors:** Khiem Quang Do, Truc Thanh Thai, Viet Quoc Lam, Thuy Thu Nguyen

**Affiliations:** 1 Department of Periodontics, Faculty of Dentistry, University of Medicine and Pharmacy at Ho Chi Minh City, Ho Chi Minh City, Vietnam; 2 Department of Medical Statistics and Informatics, Faculty of Public Health, University of Medicine and Pharmacy at Ho Chi Minh City, Ho Chi Minh City, Vietnam; 3 Department of Oral and Maxillofacial Radiology, Faculty of Dentistry, University of Medicine and Pharmacy at Ho Chi Minh City, Ho Chi Minh City, Vietnam

**Keywords:** AAP2017, Artificial intelligence, Automated diagnosis, Panoramic radiographs, Periodontal diagnosis

## Abstract

**Background:**

Periodontal diseases are common chronic conditions that can lead to tooth loss and systemic complications if not diagnosed and treated promptly. The 2017 classification by the American Academy of Periodontology highlights the need for effective, accurate diagnostic tools. This study aimed to develop and validate an AI-driven system for automated staging and grading periodontitis from panoramic radiographs using the YOLOv8 architecture.

**Methods:**

A total of five hundred panoramic radiographs from patients diagnosed with periodontitis were included. Radiographs were labeled and split into training (75%), validation (15%), and testing (10%) sets. Three specialized YOLOv8-based models were trained to segment the alveolar bone level, the cemento-enamel junction (CEJ), and tooth axes. Image augmentations were applied to enhance model robustness. The resulting measurements of radiographic bone loss were combined with patient information (age, smoking status, diabetes) to identify periodontitis stage and grade following the 2017 guidelines.

**Results:**

The bone level and CEJ detection models achieved high precision (0.95–0.97) and recall (0.94–0.96), reflecting strong segmentation performance. The tooth detection model achieved a precision of approximately 0.82 and a recall of 0.81. Integrating all three models enabled automated determination of periodontal stage (I–IV) and grade (A–C), with an interactive interface allowing clinicians to review and adjust outputs if necessary.

**Conclusion:**

The proposed YOLOv8-based framework accurately detects key periodontal landmarks and automates disease staging and grading. Future work should expand the dataset, refine the tooth detection model, and validate the system in clinical settings to support large-scale periodontal screening and improved patient care.

**Supplementary Information:**

The online version contains supplementary material available at 10.1186/s12903-025-07025-8.

## Introduction

Periodontal diseases, which are the most common non-communicable chronic diseases and affect billions of individuals worldwide, are major concerns in global health [1]. These diseases often have subtle clinical signs in the early stages that may not be obvious to patients (such as bleeding on probing, gingival edema, and color changes) and can result in significant consequences if untreated, including tooth loss, mastication dysfunction, and diminished quality of life. Moreover, periodontal diseases have been proven to be associated with other chronic systemic diseases, such as diabetes [2] and cardiovascular diseases [3], which further emphasizes the need for early detection and timely intervention. Also, the silent progression of periodontal diseases highlights the importance of developing more effective and accurate diagnostic methods.

Traditional diagnostic methods based on clinical examination and conventional radiographs have some inherent limitations [4]. Clinical assessment through probing every pocket depth as well as assessing bone loss through radiograph can be time-consuming, subjective, and reliant on the clinician’s experience [5]. Recently, a branch of artificial intelligence (AI), computer vision, has been widely applied to medical image analysis and has shown remarkable potential in diagnostic capacity. AI models have been proven to have the ability to reduce human errors, automate processes, save time, and improve the accuracy of medical diagnoses [6].

In 2017, following almost two decades, the American Academy of Periodontology (AAP) and the European Federation of Periodontology (EFP) proposed a new classification framework, highlighting staging and grading of periodontal diseases [7]. The staging relates to the severity, extent, and complexity of the disease, while the grading relates to disease advancement and risk factors, such as smoking and diabetes. Despite its strong scientific basis, the multifaceted nature complicates its real-world use [8]. AI-driven models, particularly convolutional neural networks (CNNs), are gaining tremendous traction as a potential solution for their efficiency.

Although AI models delivered strong performance in research settings [9], they often failed to meet multidimensional clinical needs when facing real patients. Most of the time, healthcare professionals demand diagnosis systems capable of incorporating critical anatomical details, clinical findings, and patient records to form a comprehensive diagnosis, which can be seen in the AAP 2017 guidelines. To enhance applicability, automated AI-enhanced solutions should be optimized as real-time AI frameworks with improved accuracy by integrating automated measurements of radiographic bone loss (RBL) from radiographs with patients’ records. These approaches may help to bridge the gap between AI’s computational strength and the evolving demands of modern dentistry.

Previous studies have demonstrated the potential of AI in periodontal diagnostics, particularly for detecting alveolar bone loss and classifying periodontitis stages from panoramic radiographs [9]. Recent advancements have seen various models applied to this task [10]. For example, studies using Deep CNNs have shown promise in staging periodontitis but often require significant preprocessing [11]. Hybrid methods combining deep learning with CAD systems have achieved high segmentation accuracy but were sometimes validated without an independent test set [12], potentially overestimating generalizability. While several studies have successfully used YOLO versions for detecting bone loss [13, 14], very few have extended this to a comprehensive system that automates both staging and grading by integrating essential patient-specific data (e.g., age, smoking, diabetes) as required by the 2017 AAP guidelines. Notably, research on automated grading is scarce and has predominantly relied on Natural Language Processing models to extract data from existing electronic health records [15]. However, the efficacy of such systems is contingent upon the availability of comprehensive, pre-existing clinical notes, which can limit their utility during the crucial initial stages of diagnosis and treatment planning. Our study aims to fill this specific research gap by developing an integrated framework that not only performs accurate segmentation using YOLOv8 but also incorporates these patient-level risk factors for a complete, automated diagnostic classification.

By integrating these data, we aim to create an automated, comprehensive system for staging and grading periodontitis, following the 2017 AAP guidelines. This system is designed to expedite the treatment planning process. Our research holds the potential to make periodontal disease diagnosis more efficient and establish a foundation for widespread periodontal screening programs through an AI diagnostic support system.

## Materials and methods

### Study design and participants

The sample size of 500 panoramic radiographs was selected based on rule-of-thumb guidelines for deep learning in medical imaging, which typically recommend 100–1000 images for internal validation of segmentation tasks to ensure sufficient data diversity after augmentation [16, 17]. In our study, the sample size consideration was also based on practice in previous studies. For example, Putra et al. (2025) conducted a study on automated periodontal bone loss detection, using an identical sample size of 500 annotated panoramic radiographs, with a comparable split (400 training, 50 validation, 50 testing), and achieved high accuracy in radiographic assessment [18]. Systematic reviews of AI models for periodontitis classification also report accuracy rates of 70–90% with sample sizes in this range [10].

This cross-sectional analytical study evaluated panoramic radiographs of patients aged 18 years and older who were diagnosed with and treated for periodontal disease at the Department of Oral and Maxillofacial Surgery, University of Medicine and Pharmacy at Ho Chi Minh City (UMP HCMC). The radiographs were taken during the patient’s treatment between 2018 and 2024 to assess the periodontal condition using the department’s Dentsply Sirona X-ray machine. Radiographs were captured at an operating setting of 85 kV/7 mA with a field of view (FOV) of 17 × 17 cm. All selected radiographs were evaluated and approved by an experienced oral and maxillofacial radiologist to ensure the quality and suitability for the study; radiographs were excluded if they were of insufficient quality, showing blurring, improper patient positioning, or artifacts from movement during the capture. Radiographs from patients under 18 years (pediatric radiographs), or those exhibiting abnormalities in tooth number, shape, or bone morphology, or radiolucent/radiopaque jawbone lesions, were also excluded; additionally, radiographs affecting the labeling of key anatomical landmarks such as CEJ and bone level, including those with orthodontic brackets, dental implants, fixed partial dentures, or single tooth crowns, were not included. Radiographs that did not affect the anatomical landmarks, such as those with root canal-treated teeth, were included, and radiographs with at least one tooth were selected to capture varying degrees of tooth loss. This research was conducted with ethical approval from the Institutional Review Board under protocol number IRB-VN01002/IORG0008603/FWA00023448, dated November 13, 2023 (approval number 1116/HĐĐĐ-ĐHYD). Since data collection was retrospective and based on panoramic radiographs, with no direct patient contact, informed consent was waived.

### Study procedures

The study’s methodology was structured into three sequential phases. First, a suitable dataset of radiographs was sourced and selected. Second, a gold standard was established through manual expert annotation. Finally, the AI models were developed and validated against this standard. The workflow was as follows:

1. Image sourcing and selection: A total of 500 panoramic radiographs were retrospectively selected from the patient database at the Department of Oral and Maxillofacial Surgery, UMP HCMC. Panoramic radiographs were selected by an experienced radiologist according to established inclusion and exclusion criteria.
2. Manual assessment and gold standard Creation: The selected radiographs were manually annotated by the same radiologist, who has 10 years of experience, using the LabelMe software. Key anatomical landmarks, including the alveolar bone margin, the cemento-enamel junction (CEJ), and tooth axes, were meticulously outlined. This complete set of expert manual annotations was established as the “gold standard” reference for all subsequent AI model development and evaluation.
3. AI model development and comparison: The gold standard dataset was partitioned into training (75%), validation (15%), and testing (10%) sets. The AI models were then trained using this data to perform the same segmentation tasks as the human expert. Finally, the models’ performance was quantitatively evaluated against the gold standard annotations using the independent test set, serving as a direct comparison of the AI’s ability to replicate the manual assessment.

### Data collection and labeling

**Fig. 1:**
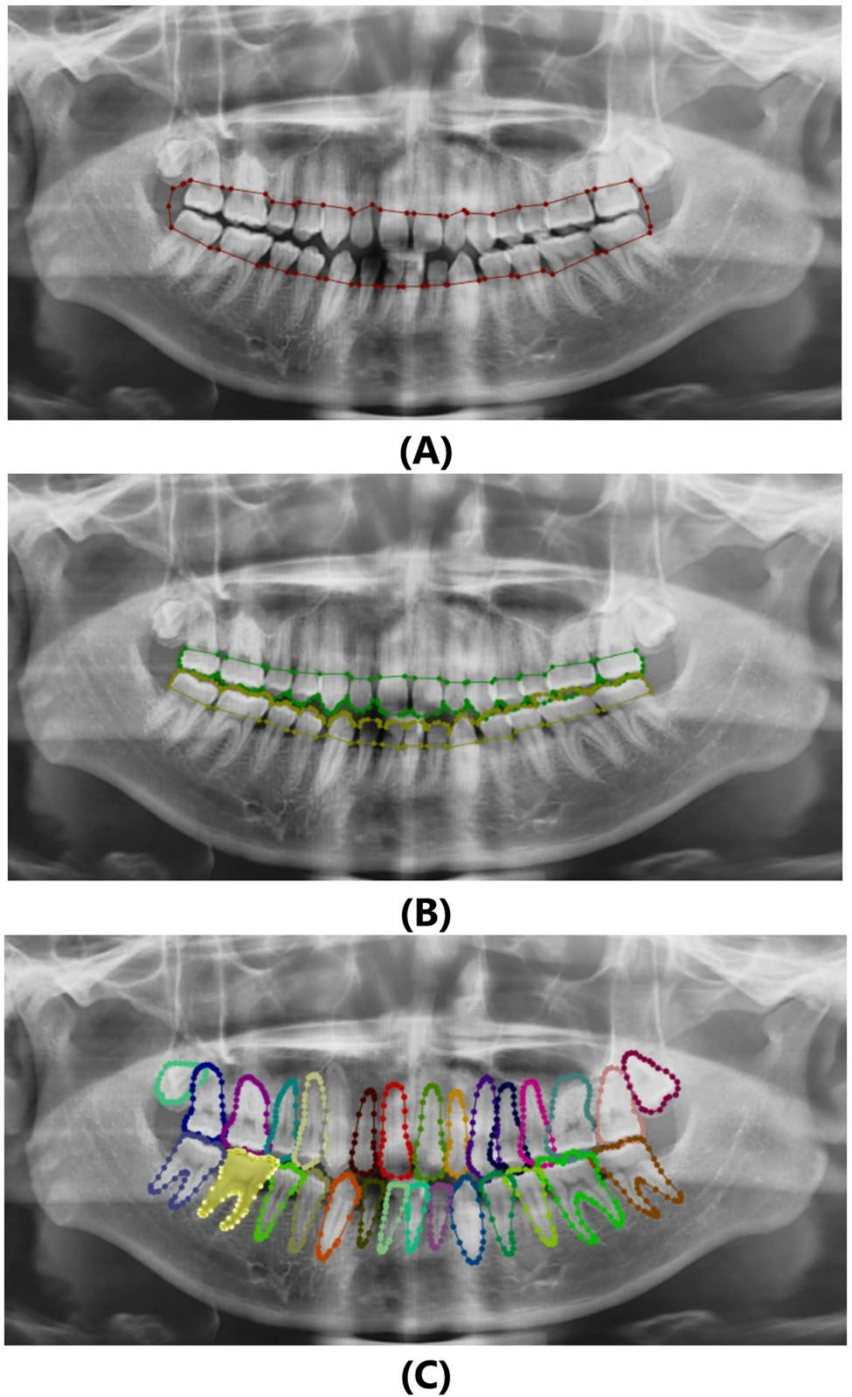
Image showing bone level (**A**), CEJ (**B**), teeth (**C**), labeled using LabelMe

Patients’ radiographs obtained from the department’s imaging machine via the SIDESIX software are stored as JPEG format. The image files were then used in the labeling step without any further preprocessing. Image data labeling was performed using the LabelMe tool by a 10-year experienced radiologist for object segmentation tasks and Labelme2yolo for data conversion. Depending on the training objective, each model had a different labeling schema. One label was used as an outline for bone detection (Fig. 1A). To detect CEJ, two labels were used (Fig. 1B). For tooth detection, thirty-two labels were used (Fig. 1C). Each labeling data was stored in a JSON file.

### AI model development

To improve segmentation accuracy and ensure precise localization of anatomical structures, this study developed three separate models instead of a single model. By adopting this method, each model was assigned to specialized detection roles including recognition of bone level contouring, CEJ identification, and tooth axis recognition. This modular approach mirrors the clinical workflow, enhancing interpretability. It allows clinicians to review the segmentation output for each anatomical structure separately, potentially making it easier to identify the source of any model inaccuracies and refine the final assessment [19]. Utilizing YOLOv8 (version 8 of You Only Look Once) as a foundation, three models were developed to combine their outputs and determine the bone loss ratio for individual teeth. The training pipeline for these three models consisted of the following processes:

#### Dataset splitting

The dataset was randomly divided into training, validation, and testing using a 75:15:10 split. In other words, a total of 75% of the data (*n* = 375) was used for training, 15% (*n* = 75) was used for validation, and 10% (*n* = 50) was used for testing.

#### Augmentation

 With the training dataset, the augmentation pipeline was as follows: additive Gaussian noise (intensity 0–5%) was first applied to simulate real-world imperfections. Then, up to four transformations were randomly selected: blend alpha regular grid (adjusting brightness/texture), affine rotation (rotating between − 10 and 10°), gamma contrast transformation (modifying contrast between 0.5 and 1.75), brightness transformation (shifting pixel values between − 30 and 30), salt and pepper noise (adding 3% random black/white pixels), and motion blur (applied with a 10 × 10 kernel and an angle between − 45 and 45°). Each training image was augmented into five different variations, expanding the dataset and improving data diversity. For the validation set, a similar augmentation process was applied but with reduced intensity to ensure the images remained closer to real-world conditions.

#### Training step

 All models were executed in Google Colab with Nvidia A100 GPUs. Default settings for all models, including early stopping with a patience of 50, were adopted. The image size was increased to 1280 to detect finer detail critical for effective detection, and hyperparameters such as batch size were optimized in relation to GPU availability. All tasks utilized uniformly the AdamW optimizer. The main adjustable hyperparameters used for each model are detailed in Additional file 1, while all remaining settings were kept at the default values of the YOLOv8 architecture.

#### Postprocessing

To integrate the results from the three models, a post-processing pipeline was established. First, the tooth identification model generates a contour for each tooth, from which a central axis is computed that extends from the crown to the root apex. This axis then serves as a reference line, intersecting the contours produced by both the cemento-enamel junction (CEJ) detection model and the bone level detection model. Based on these intersection points, the system automatically calculates two key distances along the axis: the distance from the CEJ to the alveolar bone crest (Distance 1) and the distance from the CEJ to the root apex (Distance 2). Finally, the radiographic bone loss (RBL) percentage is calculated for each tooth using the formula: RBL(%) = Distance 1/Distance 2 × 100%. This resulting RBL value is then used to determine the periodontitis stage for each tooth according to the 2017 AAP criteria [7].

#### Staging

The severity of periodontitis for each tooth was determined based on the criteria of bone loss on radiographs according to the 2017 AAP classification [7]. Specifically, Stage I (mild) indicated bone loss of less than 15% on radiographs, Stage II (moderate) indicated bone loss ranging from 15% to 33% on radiographs, and Stages III–IV (severe) indicated bone loss of more than 33% on radiographs (Table 1). Stages III and IV were further distinguished by additional clinical criteria, and the condition of the most severe tooth determines the overall stage of periodontitis for the patient.

**Table 1:** AAP 2017 periodontitis staging based on radiographic bone loss

	Radiographic bone loss	
	**Position**	**Percentage**
Stage I	**Coronal third**	**< 15%**
Stage II		**15% to 33%**
Stage III	**Extending to mid-third of root and beyond**	**> 33%**
Stage IV		

Adapted from Tonetti et al. (2018) [20]

#### Grading

When periodontal records older than five years were unavailable, the grade of periodontitis was determined based on indirect evidence of progression using the primary criterion (employed when available), which involved calculating the percentage of bone loss relative to the patient’s age according to the 2017 AAP guidelines [7]. Grade A (slow rate) was defined by a ratio of less than 0.25, Grade B (moderate rate) was defined by a ratio of 0.25–1.0, and Grade C (rapid rate) was defined by a ratio of greater than 1.0. Once the initial grade was established, it was then modified based on the presence of risk factors. If no risk factors were present, the original grade remained unchanged. However, if a patient smoked fewer than ten cigarettes per day or had diabetes with an HbA1c level below 7.0%, the grade was upgraded from Grade A to Grade B. If a patient smoked more than ten cigarettes per day or had diabetes with an HbA1c level exceeding 7.0%, the grade was upgraded to Grade C, regardless of the initial grade (Table 2).

**Table 2:** AAP 2017 periodontitis grading based on the bone loss/age ratio

Classification	Primary Criterion (% bone loss/age)	Grade modifiers	
		Risk Factor (Smoking)	Risk Factor (Diabetes)
GRADE A (Slow progression)	< 0.25	Non-smoker	Normoglycemic/no diagnosis of diabetes
GRADE B (Moderate progression)	0.25 to 1.0	Smokes < 10 cigarettes/day	HbA1c < 7.0% in patients with diabetes
GRADE C (Rapid progression)	> 1.0	Smokes ≥ 10 cigarettes/day	HbA1c ≥ 7.0% in patients with diabetes

Adapted from Tonetti et al. (2018) [20]

### Model evaluation and validation

To robustly evaluate the stability of our model’s performance, an image-level bootstrap analysis was conducted on the independent test set (50 radiographs). This was used to assess how well the AI models generalize to unseen data relative to the reference standard established by an experienced oral and maxillofacial radiologist, who manually labeled key parameters, including alveolar bone margin (bone level), cemento-enamel junction (CEJ), and tooth axes. We performed 1,000 iterations of resampling with replacement to generate a distribution for each performance metric. For each bootstrap sample, a confusion matrix estimated true positive (TP), true negative (TN), false positive (FP), and false negative (FN) values. Key performance metrics, including precision, recall (sensitivity), specificity, F1-score, accuracy, Dice score, and Jaccard index, were computed, with 95% confidence intervals derived from these distributions to provide a reliable estimate of the model’s expected performance and its variability on unseen data. Since the final disease stage was derived from a rule-based calculation applied to these segmentation outputs, not from a direct classification model, a confusion matrix for staging was not generated. The evaluation, therefore, focused on quantifying the performance of the foundational segmentation tasks.

**Fig. 2:**
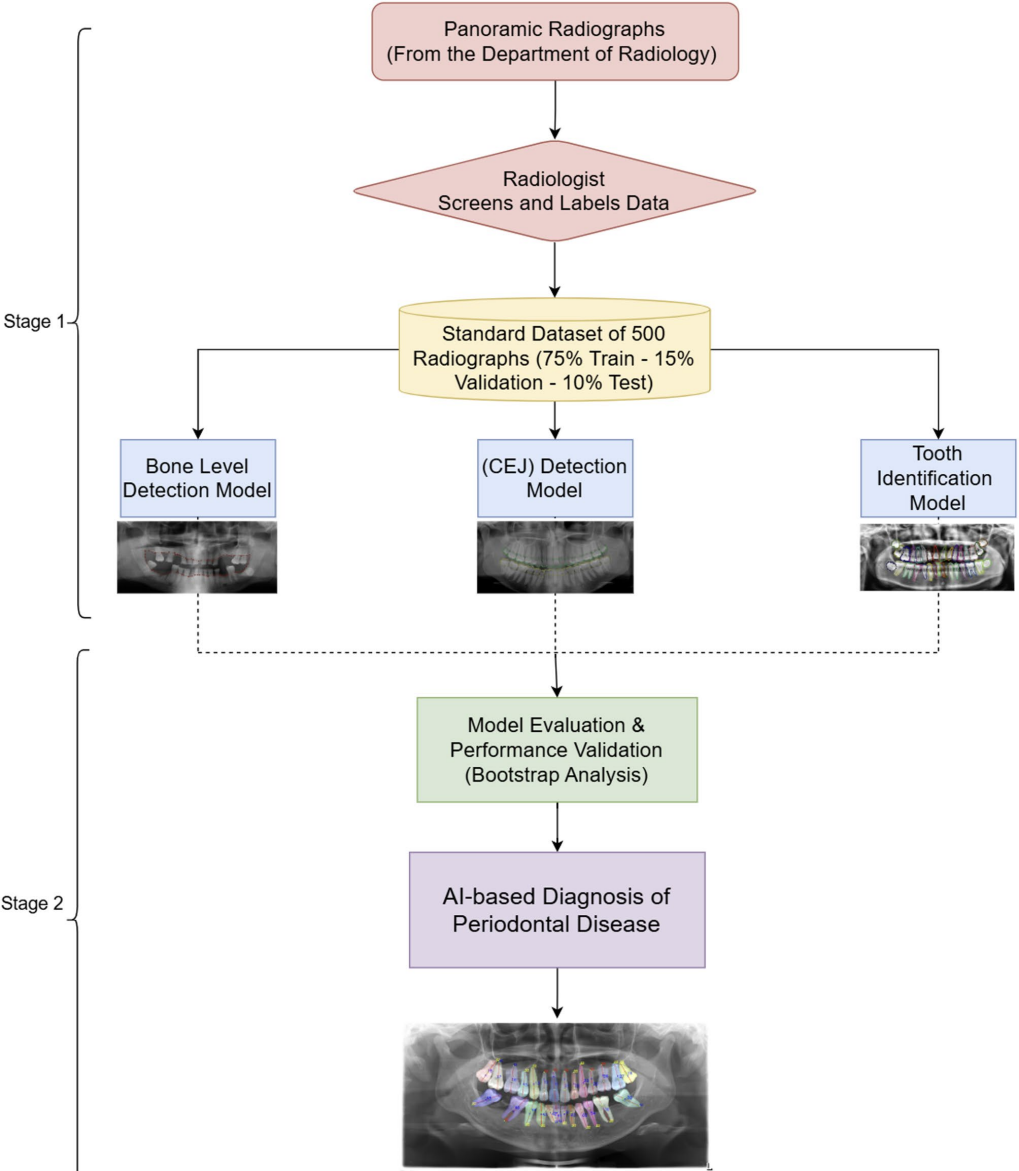
Ai-based workflow for the diagnosis of periodontitis using panoramic radiographs

These metrics quantify the level of agreement between the AI-generated segmentations and the manual annotations (the reference standard), serving as a direct comparison of the AI model’s performance against the human expert standard for this internal validation study. Other factors, including tooth loss and complexity aspects (such as furcation involvement, probing depth, vertical bone loss, and ridge defects), were not evaluated, as our focus was on radiographic bone loss (RBL) for AAP 2017 staging/grading. The study workflow is illustrated in Fig. 2.

### Development of an AI-assisted periodontal diagnosis interface

For better interpretation of the AI-based segmentation results, the final YOLOv8 model was deployed as a web-based interactive application. This setup used Gradio to allow clinicians to upload panoramic X-ray images, automatically segment relevant structures, and provide preliminary staging and grading of periodontal disease according to the AAP 2017 guidelines. Additionally, age, smoking habits, or diabetes could be included for adjusting the system’s grading.

## Results

A total of five hundred panoramic radiographs were included in this study, all of which were diagnosed with periodontitis and staged by an experienced periodontist according to the 2017 American Academy of Periodontology (AAP) classification. As shown in Tables 3 and 185 radiographs (37.0%) were categorized as Stage I, 161 (32.2%) as Stage II, and 154 (30.8%) as Stage III. A Chi-square test demonstrated no statistically significant difference across the three stages (*p* = 0.21), indicating that the distribution of radiographs among Stages I, II, and III was balanced. This balance mitigated the risk of model training bias, ensuring the model was not skewed towards predicting a more frequently represented stage.

**Table 3:** Distribution of the five hundred panoramic radiographs across three periodontal disease stages

Stage	Number of Radiographs	Percentage (%)	*p*-value
Stage I	185	37.0	0.21
Stage II	161	32.2	
Stage III	154	30.8	
Total	500	100	

Table 4 presents the performance metrics of the three models applied to detect critical anatomical structures. Bone level and CEJ detection models demonstrated remarkable performance in identifying periodontal landmarks. F1-scores achieved near-perfect accuracy, with a minor performance drop on the test set. The CEJ detection model achieved an ideal specificity score, which indicated that it correctly identified CEJ regions without false positives. Meanwhile, the bone level model maintained exceptional precision and recall (both surpassed 0.93 in the test set) suggesting strong segmentation accuracy. On the test set, the performance of both models remained high but showed a minor degradation in the validation set.

**Table 4:** Performance metrics and 95% confidence intervals of three models for determining the bone margin, identifying the CEJ, and detecting the teeth

	Precision	Recall	Specificity	F1_score	Dice score	Jaccard index	Accuracy
Based on validation dataset							
Bone level	0.97 (0.96–0.97)	0.96 (0.96–0.97)	0.99 (0.99–0.99)	0.97 (0.96–0.97)	0.96 (0.96–0.97)	0.94 (0.93–0.94)	0.99 (0.99–0.99)
CEJ	0.95 (0.94–0.95)	0.96 (0.96–0.97)	1.00 (1.00–1.00)	0.95 (0.95–0.96)	0.95 (0.95–0.96)	0.91 (0.90–0.92)	1.00 (0.99–1.00)
Teeth detection	0.90 (0.84–0.95)	0.89 (0.83–0.94)	1.00 (1.00–1.00)	0.89 (0.84–0.93)	0.89 (0.84–0.93)	0.81 (0.73–0.87)	1.00 (1.00–1.00)
Based on test dataset							
Bone level	0.95 (0.94–0.95)	0.94 (0.93–0.96)	0.99 (0.99–0.99)	0.95 (0.94–0.95)	0.95 (0.94–0.95)	0.90 (0.88–0.91)	0.99 (0.99–0.99)
CEJ	0.93 (0.92–0.94)	0.94 (0.91–0.97)	1.00 (1.00–1.00)	0.94 (0.92–0.95)	0.94 (0.92–0.95)	0.88 (0.85–0.90)	1.00 (1.00–1.00)
Teeth detection	0.82 (0.74–0.90)	0.81 (0.72–0.89)	1.00 (1.00–1.00)	0.81 (0.73–0.88)	0.81 (0.73–0.88)	0.69 (0.58–0.79)^*^	1.00 (1.00–1.00)

^*^The wider confidence interval for the Jaccard index in the tooth detection model reflects the increased complexity and variability inherent in the 32-class segmentation task (identifying each individual tooth) compared to the single-class segmentation for bone level and CEJ

In contrast, the tooth segmentation model performed at an acceptable level compared to the other models, still lagged behind the bone level and CEJ models. Specifically, when evaluated on the test dataset, it achieved a precision of 0.82 (0.74–0.90). A major reason for this discrepancy is the initial use of multi-class segmentation. When the model demanded to recognize thirty-two distinct teeth, it would be significantly more challenging than segmenting broader regions like bone level or CEJ. While the model’s accuracy shows a perfect accuracy of 1.00, the wide confidence interval for the Jaccard index (0.58–0.79) suggests fluctuation in the model’s performance for difficult tasks. This underscores the ongoing challenge of tooth segmentation due to substantial diversity in tooth shape, alignment, and positioning.

**Fig. 3:**
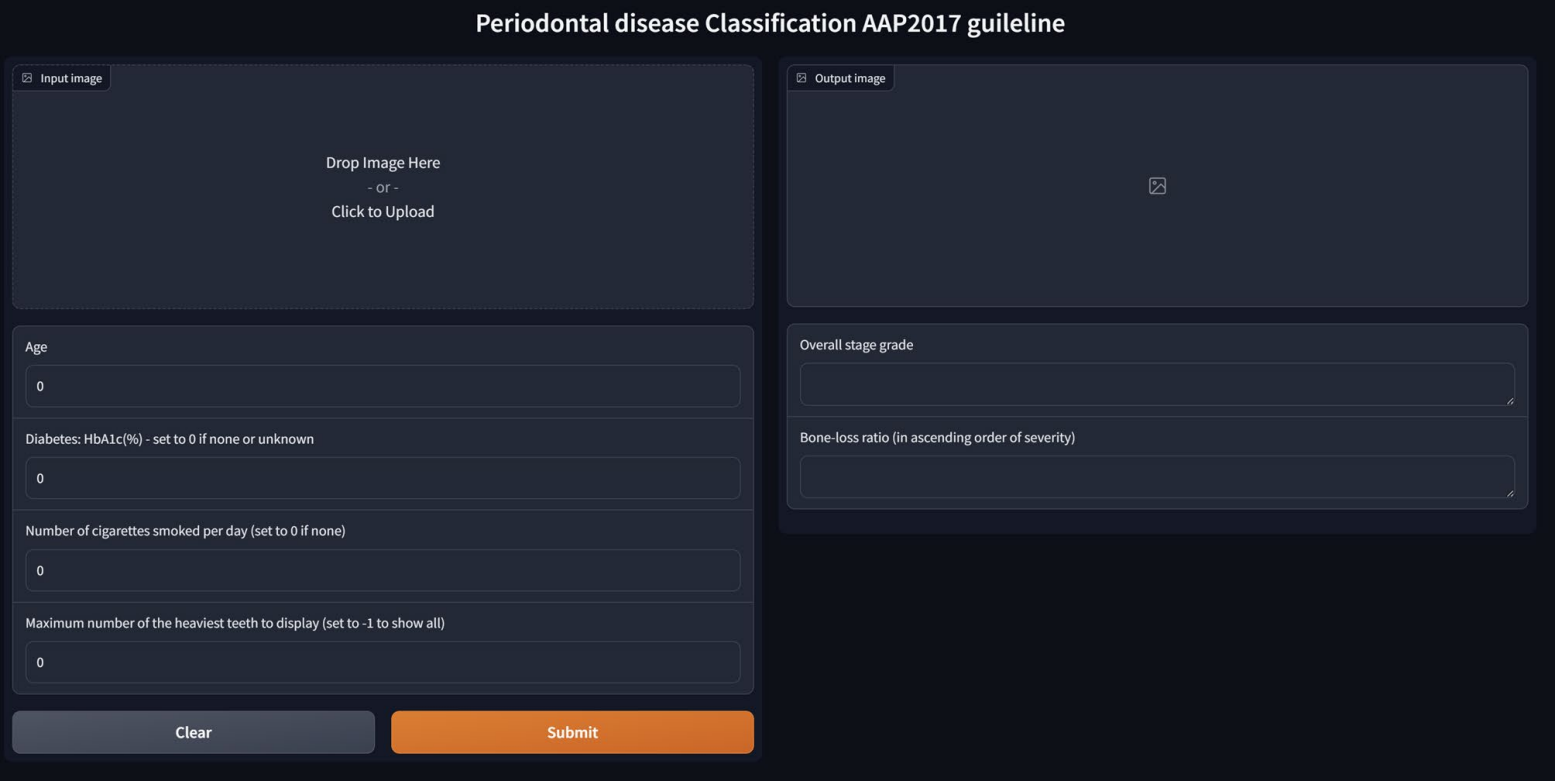
Graphical user interface of the AI-assisted periodontal disease classification system based on the AAP 2017 guidelines

**Fig. 4:**
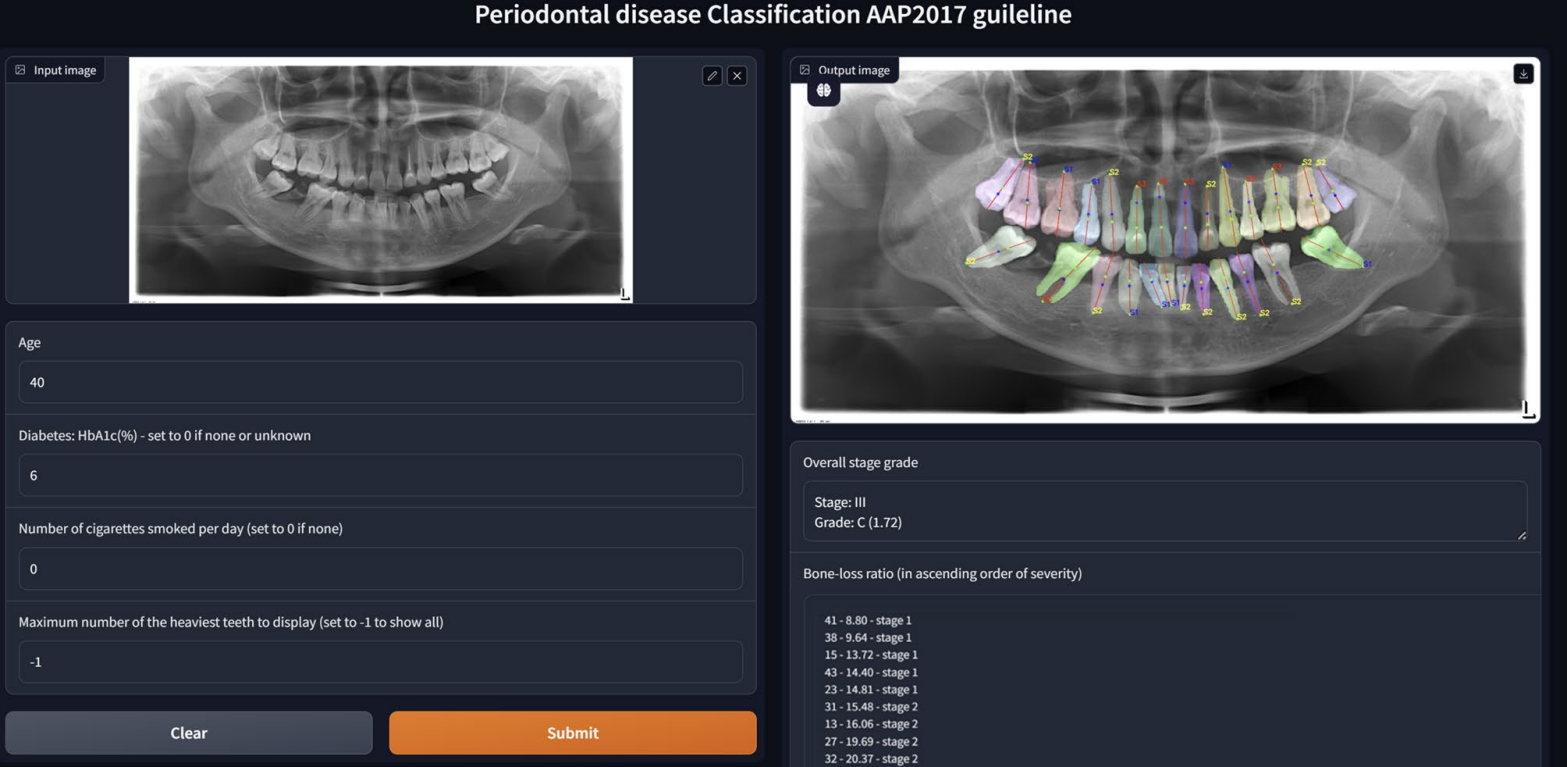
The result image shows the AI based detection of bone levels, CEJ and tooth axes

**Fig. 5:**
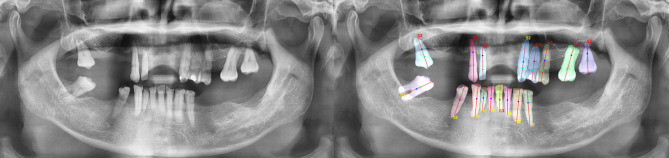
A case of underestimation of periodontal disease severity by the AI model. Tooth #23 was incorrectly staged as stage II instead of stage III

Three trained models were integrated into an automated periodontal assessment system that takes a panoramic radiograph and patient data, including age, HbA1c (%), and smoking habits, as inputs (Fig. 3). The AI calculated the bone loss ratio for each tooth, which was then used with patient data to determine the periodontal grade by applying the AAP 2017 guidelines, not through AI processing. Adjustments for diabetes or smoking were also made strictly per guideline recommendations. The system provided segmentation overlays on the radiograph, enabling clinicians to review the diagnosis and correct inaccuracies if needed (Fig. 4). While the system demonstrated successful segmentation in many cases (Fig. 4), instances of misclassification were also observed. Figure 5 illustrates such a case, where the AI model incorrectly classified the periodontitis on tooth #23 as Stage II. In contrast, the periodontist assessment based on the radiographic evidence indicated a more severe condition corresponding to Stage III, showing an underestimation of bone loss by the model.

## Discussion

Periodontal diseases pose a major public health concern worldwide, affecting a significant proportion of the population and resulting in tooth loss and masticatory dysfunction. Also, there is a well-established link between periodontal disease and major medical complications, such as diabetes [2] and cardiovascular diseases [3]. The asymptomatic progression of periodontal diseases in their early stages makes timely and accurate detection crucial. Conventional diagnostic approaches can be time-consuming and subjective, leading to inter-clinician variability in diagnoses [5]. In this context, AI, particularly computer vision, offers a paradigm shift in periodontal diagnostics by optimizing accuracy, efficiency and workflow. This study develops an AI-based framework utilizing YOLOv8 for the automated categorization of periodontitis severity, adhering to the 2017 American Academy of Periodontology (AAP) guidelines [20]. Our main findings demonstrate that the YOLOv8-based models achieved superior precision and recall, exceeding 0.95 for bone level and CEJ detection, and performing acceptably for tooth detection, enabling accurate segmentation of alveolar bone, CEJ, and tooth axes. When integrated with patient data, the model can execute staging and grading of periodontal disease.

The reliable localization of bone levels and CEJ is essential for calculating radiographic bone loss (RBL), a key indicator of disease severity [20]. In this study, the YOLOv8-based models for bone level and CEJ detection demonstrated exceptional performance, achieving precision and recall metrics exceeding 0.95 and specificity approaching 1.0 on both validation and test datasets. These strong evaluation results indicate the model’s capabilities to precisely segment critical anatomical structures even in the presence of inconsistencies in X-ray clarity and anatomical complexities. The robustness of the YOLOv8 architecture, originally designed for object detection, was effectively adapted for segmentation tasks, enabling precise landmark detection crucial for RBL calculations [18]. Differing from standard object detection techniques, YOLOv8 implements a anchor-free segmentation strategy to minimize reliance on fixed bounding boxes and improve flexibility across different images [21]. Studies comparing YOLOv8 with Mask R-CNN, which is built for segmentation, in dental imaging and agricultural segmentation have shown its superior accuracy and computational efficiency, with inference speeds nearly 30% faster than two-stage models [21, 22]. Compared to previous studies, such as Jiang et al. (2022) [11], which utilized UNet and YOLO-V4, reported lower precision (e.g., 0.77 for overall staging), our YOLOv8 approach showcases superior segmentation accuracy. Chang et al. (2020) utilized a Mask R-CNN-based approach that integrated deep learning with CAD processing and achieved a Dice coefficient of 0.93 for bone level detection and 0.91 for CEJ [23]. However, their evaluation was based entirely on a validation dataset lacking an independent test dataset, reducing its ability to generalize. By comparison, our model attained higher Dice scores (0.95 for bone level and 0.94 for CEJ) while ensuring consistent performance across multiple dataset evaluations. The minor accuracy reduction observed in the test set (e.g., precision decreasing from 0.97 to 0.95 for bone level detection) is common in AI frameworks and implies effective real-world applicability and strong generalizability. This validation on an independent test set provides stronger evidence of generalizability, a component that was not reported in the study by Chang et al. (2020).

Compared to bone and CEJ detection, the tooth detection model exhibited lower accuracy. Unlike bone and CEJ detection, tooth segmentation involves the complex task of classifying thirty-two separate labels. Our model achieved a Dice score of 0.89 for tooth detection on the validation set, which is slightly lower than the 0.91 reported by Chang et al. (2020). Unlike their study, which was limited to validation results, our model was further evaluated on an independent test dataset, where the Dice score dropped to 0.81. The decline in Dice score emphasizes why separate test set evaluations are critical, as relying on validation data can lead to overestimation of performance. Another key difference is that our model segments individual teeth, whereas Chang et al. (2020) grouped all teeth under a single label, simplifying their segmentation task. Although this approach improves the Dice score, it may limit the real-life application in detailed dental diagnostics.

A comparison with the recent work by Jundaeng et al. (2025) [14], which also utilized a YOLOv8-based segmentation approach on panoramic radiographs, further clarifies our framework’s contributions. Despite a smaller dataset, our specialized models for bone level and CEJ detection achieved higher F1-scores (0.95 and 0.94, respectively) than their single combined model (0.90). While our models showed superior precision and specificity, this higher F1-score is particularly significant as it represents a better balance between precision and recall, a key indicator for segmentation tasks. Similarly, our tooth detection model’s F1-score was slightly higher (0.81 vs. 0.80), a notable result given its increased task complexity. Our model identifies thirty-two distinct tooth classes, a significantly more challenging and clinically relevant objective than their single class “tooth” label, which is critical for distinguishing individual teeth, especially in cases of crowding. Furthermore, our modular framework of three separate models enhances clinical interpretability, allowing clinicians to independently review and adjust outputs for each anatomical structure. This facilitates more precise diagnostics compared to a system where key landmarks are combined.

Additionally, the YOLOv8 model’s real-time processing capability makes it well suited for clinical applications [24], enabling faster decision-making in automated periodontal assessments. Although these issues exist, our tooth recognition system’s impact on computerized disease staging was demonstrated by correctly integrating high-accuracy bone and CEJ detection. However, the variation in accuracy highlights a potential avenue for optimization. It might be mitigated using refined AI structures and an expanded number of labeled images. As indicated by Shon et al. (2022), who obtained higher segmentation accuracy for teeth via YOLOv5, despite relying on a more extensive dataset [25].

The strong precision and consistency of our models emphasize their applicability in clinical practice. By enabling AI-driven identification of critical dental landmarks and linking this information to clinical documentation, our system can help to minimize assessment duration while enhancing objectivity, therefore promoting consistency across clinicians. This is highly valuable in low-resource healthcare systems, where there is a shortage of experienced periodontists. The capability to classify periodontal disease severity following the AAP 2017 framework improves therapeutic decision-making and enables timely clinical response, potentially mitigating disease progression and systemic complications. From a clinical perspective, our AI system can act as a clinical decision aid, allowing healthcare professionals to effectively visualize bone loss ratios and stage disease efficiently and consider patient-specific characteristics. This tool may hold promises for large-scale periodontal screening programs; help detect high-risk individuals at an early stage and optimize resource allocation. Additionally, its capacity to monitor disease progression over time could guide continuous patient monitoring strategies, aligning with global health initiatives to reduce periodontal disease burden [1].

Despite its strengths, this study has limitations. First, a sample set of five hundred dental X-rays is somewhat restricted in size, possibly affecting the model’s generalizability across diverse populations and imaging conditions. Second, the lower performance in individual tooth segmentation indicates a need for further improvement, particularly in handling anatomical complexities. Third, dependence on 2D imaging and clinical history omits complementary radiographic methods, such as intraoral scans or 3D imaging, which might improve diagnostic precision. Finally, in our study, model evaluation was mainly based on internal validity and practical testing in clinical environments is yet to be conducted, which is crucial for establishing its diagnostic reliability in diverse clinical scenarios.

Furthermore, our system employs a hybrid approach where the outputs from the AI segmentation models are fed into a rule-based system derived from the AAP guidelines to determine the final staging and grading. While this enhances clinical interpretability, it has inherent limitations. The system’s accuracy is critically dependent on the performance of the upstream segmentation models; any inaccuracies in detecting the CEJ or bone level will directly lead to incorrect staging and grading. This rule-based post-processing step does not suffer from overfitting in a traditional machine learning sense, as the rules are fixed clinical guidelines. However, it can lead to inconsistent results if the input data from the AI is variable, especially in borderline cases. This trade-off between an automated, interpretable, rule-based system and a potentially more robust end-to-end deep learning model requires further investigation in future work.

Upcoming refinements should focus on increasing the dataset by incorporating varied patient groups with different scenarios, strengthening the system’s generalization. Another good consideration is refining the tooth detection model with advanced architecture (e.g., transformers) or additional data could elevate performance. The next critical step will be developing a user-friendly interface for clinical deployment, with subsequent trials to measure practical outcomes.

## Conclusion

The AI models using YOLOv8 for automated staging and grading periodontitis from panoramic radiographs showed high precision and recall for bone level and CEJ detection, with acceptable tooth detection. Integrating these results with patient data, including age, smoking, and diabetes condition, the system determined disease stage and grade per 2017 AAP guidelines, showing potential to improve diagnostic accuracy and efficiency over traditional methods.

## Supplementary Information

Supplementary Material 1: Table S1, containing the training hyperparameters for the YOLOv8-based models.

## Data Availability

The datasets used and/or analyzed during the current study are available from the corresponding author on reasonable request.
