# Supplementary material for: Development and validation of artificial intelligence models for automated periodontitis staging and grading using panoramic radiographs
Source: BMC Oral Health. 2025 Oct 14;25:1623. doi: 10.1186/s12903-025-07025-8 (PMC12522615; doi:10.1186/s12903-025-07025-8)
Supplement: Supplementary file 1 — Supplementary Material 1: Table S1, containing the training hyperparameters for the YOLOv8-based models. [file 12903_2025_7025_MOESM1_ESM.docx]

**Table S1.** Training Hyperparameters for YOLOv8-Based Models.

| Model | Epoch | Image size | Batch size | Learning Rate | Optimizer |
| --- | --- | --- | --- | --- | --- |
| Bone Level Detection | 200 | 1280 | 16 | 0.01 | AdamW |
| CEJ Detection | 198 | 1280 | 12 | 0.01 | AdamW |
| Tooth Identification and Axis Detection | 259 | 1280 | 8 | 0.01 | AdamW |
